# Chicago Pathological Society

**Published:** 1888-11

**Authors:** 


### CHICAGO PATHOLOGICAL SOCIETY.

Stated meeting, October 16, President I. N. Danforth, M. D., in the chair.

The session opened with a discussion on Professor Lydston's paper, "Syphiloma of the Tongue," which was read at a previous meeting.

The PRESIDENT said that a section of the specimen showed it to be a mixed sarcoma, with no indications of malignancy.

Dr. WESCOTT asked what Dr. Lydston based his opinion upon in believing that the growth was cancerous.

Dr. COPELAND said that from the general appearances of the growth Dr. Lydston thought it might have degenerated into cancer.

Dr. W. H. HAYMAN asked whether such a growth ever became malignant.

The PRESIDENT replied, "Yes; when it changes its cell type. It then becomes atypical."

Professor A. E. HOADLEY had not heard or read the paper; but the degeneration of such a growth from a benign to a malignant condition, he said, is not regarded as being well proven—that is, assuming that there are embryonal tissue-elements in the make-up of a malignant tumor. It is believed now that there is no such thing as a syphiloma ever becoming a carcinoma, or an epithelioma ever developing flat or spindle-shaped cells without the spindle-shaped cells originally existed as a basis for the development of the tumor. A syphiloma, however, might be an exciting cause for the development of carcinoma, but if it exists without the primitive embryonal cell-structures, it can never degenerate into a malignant tumor. This is yet a question in the minds of the profession.

The committee, composed of Drs. Dion, W. H. Hayman, and J. J. M. Angear, previously appointed to examine the literature on the subject of superfœtation, and to report on Dr. Murdock's case, reported conjointly as follows:

Churchill concludes:

1. That the theory of superfœtation is unnecessary to explain the birth of a mature fœtus and blighted ovum; of a mature and immature fœtus, born together or within a month of each other, or of fœtuses of different colors, as they may reasonably be supposed to be the product of one act of generation, or of two nearly contemporaneous.

2. That in cases of double uterus it is possible for a second conception to take place, and (judging from the subsequent birth of the second child, in the only case on record) at a later period than the first.

3. That in the remaining cases, where one mature child succeeded the birth of another after a considerable interval, we have no proof of a double uterus in any, and positive proof that in one case it was single, and that to the explanation of these cases no theory as yet advanced is adequate; that of superfœtation being opposed by physical difficulties, insurmountable in our present knowledge.

Simpson, Duncan, Tyler Smith, and others, deny that fecundation is prevented by the condition of the uterus in the early months of gestation. Tyler Smith has shown the mucous plug to be different only in quantity and not in kind to that which occupies the cervix in the unimpregnated state, and will not prevent passage of spermatozoa. Neither does any obstacle occur to the passage of a second ovum when the uterus is already occupied by one. Inasmuch as neither of the layers of the decidua in the first month of pregnancy pass over the orifices of the Fallopian tubes or cervical canal, until the third month the ovum with its envelopes is attached to a small portion only of one of the parietes of the uterus, leaving every other portion of the decidua vera free for the reception and development of a second ovum. "The infrequency depends," says Tyler Smith, "upon the absence of perfect ovulation during pregnancy, and not to mechanical impediment."

Playfair quotes Bonnar's case from the British peerage, in which a child was born September 12, 1849, and the mother gave birth to another January 24, 1850, an interval of 127 days. Subtracting fourteen days, which Dr. Bonnar assumes to be the earliest possible period at which a fresh impregnation can occur after delivery, the gestation is reduced to 113 days, less than four calendar months. As both survived, the second child could not possibly have been the result of a fresh impregnation after the birth of the first; nor could the first have been a twin prematurely delivered, for if so, it must have only reached a little more than the fifth month and would not have lived.

Cazeau, in 1853, obtained a placenta from a woman who was delivered at full term of a fully-developed child. It had two amniotic bags; one belonged to the living child and presented no unusual appearances; the other, much smaller, contained barely a trace of fluid, but a mummified fœtus the size of one of four months' development. The dead fœtus may irritate the uterus, bring on contractions, and be expelled, the other remaining to full development.

Hamilton (1792) says: "Soon after impregnation the uterine orifices become entirely clogged up by a thick, viscid gelatine. The internal cavity is also lined by the external membrane of the ovum, which attaches itself to the whole internal surface of the womb. The Fallopian tubes also become flaccid, and, as gestation advances, are removed from the ovaries and can not convey the ovum. From these and other reasons the doctrine of superfœtation is now generally exploded."

Burton (1751) attended a woman who, in two subsequent pregnancies, had the remains of a fœtus and integuments attached to the placenta.

Glison reports the case of an Indian woman, of Brazil, who gave birth within short periods to an European, an Indian, and an African child, and says the second child must be born three months later than the first in order to be called superfœtation.

Barnes says physiologists must first prove that ovulation takes place during pregnancy, before the doctrine of superfœtation can be substantiated.

Ross, of Brighton, reports a case (*The Lancet* August, 1871), in which a patient miscarried of twins on July 16, 1870. Fifteen weeks later, October 31, she was delivered of a healthy child, but this case was one of double uterus, each side of which had been impregnated. The patient had given birth to six children at term, nothing remarkable having been observed in her labors.

Eisemann reports the case of a woman who was delivered of a second child 140 days after the first, both having been mature.

Moseley, in his work on Tropical Diseases, reports a case of a negro woman who was delivered of two children at one birth, one negro, the other mulatto. She told the physician attending her that a white man on the estate was the father of the mulatto. Cases of the same kind and in the same circumstances have been published by Bouillon, Dewees, Crotti, Guerarde, Dunglison.

Desgranges, of Lyons, speaks of a woman, who was delivered on January 20, 1780, of a seven months' fœtus, and on July 6, 1780, five months and sixteen days later than the former birth, she was delivered of a second child which had apparently reached its full time.

Bigard, of Strasburg, reports the case of a woman, aged 37, who was delivered of a lively child on the 30th of April, the lochia and milk were soon suppressed. On the 17th of September of the same year (year not mentioned), about four and a half months after the first delivery, she brought forth a second apparently mature and healthy child. On the death of the woman the uterus was found to be single.

Maton reports the case of an Italian lady, married to an Englishman, who was delivered of a male child at Palermo, November 12, 1807. On the 28th of February, 1808, not quite three calendar months after the preceding accouchment, she was delivered of a second male child. Other similar cases are quoted by Beck, Velpeau, and Cuming.

Madame Boivin reports a case of a woman aged 40, who gave birth to a female child on the 15th of March, 1810, weighing about four pounds. As the abdomen remained large, Mde. Boivin introduced her hand, but could find nothing in the uterus; her examination led her to suspect that there was another fœtus, either extra-uterine, or contained in a second cavity in the womb. On the 12th of May, of the same year, a second female infant was born weighing not more than three pounds, feeble and scarcely able to live.

Buffon reports a case of a female at Charleston, South Carolina, who was delivered in 1714 of twins, within a very short time of each other. One of those was black, the other white. This circumstance led to an inquiry, when the woman confessed that on a particular day, immediately after her husband had left his bed, a negro entered her room and compelled her to gratify his wishes, under threats of murdering her. The second copulation took place immediately after the first.

The above case is selected to illustrate the subject of super-fecundation or superconception, but it is erroneously reported as one of superfœtation.

Many of the cases reported as cases of superfœtation are not proven to be such beyond a doubt. Some are not well reported, showing great carelessness on the part of the reporter by omitting to state important points. The following are a few cases which resemble more or less the case which was reported by Dr. Murdock.

Dr. Cairns exhibited to the Obstetrical Society of Edinburgh, June 9, 1869, a beautiful specimen of a blighted twin which had been removed from a patient immediately before the birth of a living child at full term. The blighted fœtus had been arrested in its development, molded into the form of the uterine walls, and retained, but owing to the membranes being entire it had not become putrid (Ed. Med. Journal, August, 1869).

Dr. Flecken relates a case in which a powerful woman was delivered of a strong living and full-timed child. After the expulsion of a very large placenta, another compact, fleshy placenta in partial connection with the former, followed. The accompanying membranes contained a six-months' fœtus, the body of which had been compressed so flat that the broadest part of the cranial and thoracic region did not exceed five lines. The bones of the cranium lay separated from each other, the nasal bones projected as sharp points, and the broken malar bones penetrated the skin. While in the sixth month of her pregnancy the woman had fallen down stairs (Med. Times and Gaz. March, 1862).

Dr. Picket, of Great Barrington, Massachusetts, relates the following case, which is of some interest in medical jurisprudence: I was called to Mrs. R., of Stockbridge, whom I found in labor, which lasted about six hours. This, for her, was rather severe, but she was safely delivered of a large, healthy child, apparently at full time. While examining for the placenta, I discovered something had apparently ossified. The placenta soon passed off and with it this apparently foreign substance, which proved to be a dead and partially decomposed fœtus, of about four months.

Dr. Wilson reports the following case to the Glasgow Medical Society: Mrs. T., on the 21st of November last, after a short and easy labor, was delivered of a living child. She menstruated for the last time about the beginning of March. When three months or thereby advanced in her pregnancy, she had twice in succession a considerable sanguineous discharge per vaginam accompanied with slight uterine pains. She, however, paid little attention to these symptoms at the time, as they did not occasion any anxiety or alarm. The placenta on examination seemed generally healthy, with the exception of a few small indurated points of a tuberculous character, scattered over various parts of its extent. Toward the margin of the placenta was found a small fœtus closely enveloped in a yellowish fat-like mass, which lay in a groove or sulcus formed in the placenta by the overlapping or bulging over of a portion of the fœtal surface. There can be no doubt, I think, this was primarily a case of twin conception, while one was blighted at or near the third month, and the other carried to the eighth, when both were nearly simultaneously expelled from the uterus.

In the *British and Foreign Medico-Chirurgical Review* for October, 1854, Dr. Thielman relates the following case of superfœtation: In July, 1852, a peasant woman became pregnant the third time; menstruation appeared twice after conception. On the 26th of March, 1853, the pains appeared and the next morning she was delivered of a girl, small but living; the after-birth came away normally. The lochia ceased in a few hours. The secretion of milk was so scanty that the child could not be supported by it. Eight days after delivery the woman returned to her household duties, but she felt the movement of a second child in her left side. On the 18th of May, that is, fifty-two days after the birth of the first child, pains came on, and the birth of a second girl, somewhat smaller, followed. From this time the secretion of milk went on so freely that both children derived sufficient nourishment.

Dr. Atchinson communicated to the Obstetrical Society of Edinburgh a case which is supposed to be an instance of superfœtation. The subject of the case was a well-formed woman, aged 30, who had been born and brought up in India, had borne two children at full period of gestation, and then followed two miscarriages; had lately been suffering a good deal from fever; her general health not good at present; system much relaxed from debility, caused chiefly by the prevalent great heat of the season. On examination I found that after a short and easy labor the child was born and the placenta discharged. The uterus was fairly contracted. On examining the child I found it to be a case of premature birth at the seventh month, which corresponded with the mother's statement. The child did not survive over a few hours. I then examined the placenta, to which, much to my astonishment, was attached a large sac. This, on closer inspection, proved to be another set of membranes in an entire state, containing another fœtus; the placenta of this last being incorporated into that of the first-born fœtus, much smaller, but with a distinct mark of union till existing. At one point the membrane of both the fœti were inseparably united, and those of the smaller fœtus were much more dense in structure and less transparent than those of the larger fœtus. The fœtus contained in the sac (which was not opened) seemed from its size and development to have completed nearly its fourth month of utero-gestation.

Dr. ANGEAR, after quoting at great length the literature upon superfœtation, closed by saying: We have found no recorded cases precisely like that of Dr. Murdock, but several similar. In Dr. Murdock's case let us suppose that the blighted fœtus had not been found, then all who knew anything of the case would have said that there had been an abortion, or there had been no conception previous to the present gestation. Suppose, again, that we had never known anything about the symptoms of a miscarriage, and the blighted fœtus had been found, then we should have come to the conclusion that it was simply a case of twins with one of the foeti dying early in fœtal life and retained several months in utero as in the examples given above. The evidence that there had been a miscarriage is not positive. It is simply negative. No one saw any foetus; therefore what positive evidence is there of a miscarriage; but does that prove that there was no miscarriage? If taken for granted that there was a miscarriage, then we have a case of double conception or super-conception (not superfœtation), or twins, with one of the fœti blighted and retained in utero and expelled with the living and viable child.

